# Cognitive‐motor interference during goal‐directed upper‐limb movements

**DOI:** 10.1111/ejn.14168

**Published:** 2018-10-20

**Authors:** Paulina J. M. Bank, Johan Marinus, Rosanne M. van Tol, Iris F. Groeneveld, Paula H. Goossens, Jurriaan H. de Groot, Jacobus J. van Hilten, Carel G. M. Meskers

**Affiliations:** ^1^ Department of Neurology Leiden University Medical Center Leiden The Netherlands; ^2^ Department of Human Movement Sciences Faculty of Behavioural and Movement Sciences Amsterdam Movement Sciences VU Amsterdam Amsterdam The Netherlands; ^3^ Rijnlands Rehabilitation Center Leiden The Netherlands; ^4^ Sophia Rehabilitation Den Haag The Netherlands; ^5^ Department of Rehabilitation Medicine Leiden University Medical Center Leiden The Netherlands; ^6^ Department of Rehabilitation Medicine Amsterdam Movement Sciences VU University Medical Center Amsterdam The Netherlands

**Keywords:** attention, cognitive‐motor interference, dual‐task, Parkinson's disease, stroke

## Abstract

Research and clinical practice have focused on effects of a cognitive dual‐task on highly automated motor tasks such as walking or maintaining balance. Despite potential importance for daily life performance, there are only a few small studies on dual‐task effects on upper‐limb motor control. We therefore developed a protocol for assessing cognitive‐motor interference (CMI) during upper‐limb motor control and used it to evaluate dual‐task effects in 57 healthy individuals and two highly prevalent neurological disorders associated with deficits of cognitive and motor processing (57 patients with Parkinson's disease [PD], 57 stroke patients). Performance was evaluated in cognitive and motor domains under single‐ and dual‐task conditions. Patterns of CMI were explored to evaluate overall attentional capacity and attention allocation. As expected, patients with neurological deficits showed different patterns of CMI compared to healthy individuals, depending on diagnosis (PD or stroke) and severity of cognitive and/or motor symptoms. Healthy individuals experienced CMI especially under challenging conditions of the motor task. CMI was greater in PD patients, presumably due to insufficient attentional capacity in relation to increased cognitive involvement in motor control. Although no general increase of CMI was observed in stroke patients, correlation analyses suggested that especially patients with severe motor dysfunction experienced CMI. Clinical ratings of cognitive and motor function were weakly associated with CMI, suggesting that CMI reflects a different construct than these unidimensional clinical tests. It remains to be investigated whether CMI is an indicator of difficulties with day‐to‐day activities.

**Abbreviations**
ANOVA: analysis of variance
CCSS: combined clinical severity score
CMI: cognitive‐motor interference
DTE: dual‐task effect
FM‐UE: fugl‐meyer upper extremity scale
MDS‐UPDRS‐III: section III (motor examination section) of the Movement Disorder Society version of the Unified Parkinson's Disease Rating Scale
MoCA: Montreal Cognitive Assessment
*P*_C_: performance on the cognitive task [%s^−1^]
PD: Parkinson's disease
*P*_M_: performance on the motor task [%s^−1^]
SCOPA‐COG: SCales for Outcomes in PArkinson's disease‐COGnition

## 1 INTRODUCTION

Unidimensional clinical tests for cognitive function and motor function may underestimate impairments of daily life activities. These activities typically require adequate interaction with the environment and often involve the simultaneous performance of two or more tasks (such as walking and talking, or writing while talking on the phone). Competing attentional demands can lead to decrement in performance, especially when the attentional demand of one or both tasks is high or attentional capacity is reduced. Interference may thus be disproportionately great in neurological conditions that are associated with deficits of motor and/or cognitive processing, such as Parkinson's disease (PD; Kelly, Eusterbrock, & Shumway‐Cook, 2012), multiple sclerosis (Leone, Patti, & Feys, 2015), Alzheimer's disease (Camicioli, Howieson, Lehman, & Kaye, 1997), and stroke (Plummer et al., 2013).

The relative change in performance associated with “dual‐tasking” is referred to as dual‐task interference or the dual‐task effect (DTE; Plummer & Eskes, 2015). In research and clinical practice, DTE is often only quantified in the motor domain (e.g., decrease in walking speed) as an index of automaticity of motor control, without considering (changes in) performance on the cognitive dual task. To better understand cognitive‐motor interference (CMI) and to be able to evaluate changes in response to treatment, it is critical to assess performance in both the cognitive and motor domain under single‐ and dual‐task conditions (Plummer & Eskes, 2015; Rochester, Galna, Lord, & Burn, 2014). Evaluation of DTEs in both domains does not only provide insight into cognitive and motor function separately, but also contributes to understanding of CMI in terms of attentional capacity (i.e., total DTE in both domains) as well as attention allocation (i.e., task prioritization) and (Plummer & Eskes, 2015; Plummer, Villalobos, Vayda, Moser, & Johnson, 2014; Plummer et al., 2013).

To date, research and clinical practice have mainly focused on the effects of a cognitive dual‐task (e.g., counting backwards or word naming) on highly automated motor tasks such as walking or maintaining balance (for reviews see Amboni, Barone, and Hausdorff (2013); Plummer et al. (2013)). Despite the potential importance for daily life performance, there are only a few small studies on DTEs during upper‐limb motor control, which is assumed to be more cognitively driven and thus less automated than gross motor activities such as walking (Alberts et al., 2008; Broeder et al., 2014; Frankemolle et al., 2010; Houwink, Steenbergen, Prange, Buurke, & Geurts, 2013; Mills et al., 2015; Pradhan, Scherer, Matsuoka, & Kelly, 2011; Van Impe, Coxon, Goble, Wenderoth, & Swinnen, 2011).

In this study, we therefore developed a protocol for evaluating patterns of CMI during simultaneous performance of a cognitive task and an upper‐limb motor task. We used it to evaluate DTEs in both the motor and cognitive domain in healthy individuals and in two highly prevalent neurological conditions associated with deficits of cognitive and motor processing (PD and stroke). These distinct patient groups were chosen as a generalized “proof of concept” because they were expected to show increased levels of CMI and because the considerable variation in severity of cognitive and motor impairments within these patient groups would allow evaluation of the association between the severity of cognitive and/or motor impairments and (patterns of) CMI.

The cognitive task consisted of the auditory Stroop task (Cohen & Martin, 1975), a time‐critical task requiring continuous attention, which has previously proved successful in eliciting CMI even in healthy individuals (e.g., Weerdesteyn, Schillings, Van Galen, & Duysens, 2003). The motor task involved goal‐directed upper‐limb movements to control a virtual mouse presented on a LED TV and to collect virtual pieces of cheese (targets) as fast as possible while avoiding a virtual cat (obstacle). Single‐task performances as well as DTEs in both the cognitive and motor domain were compared between healthy individuals, PD patients with varying degree of cognitive and motor symptoms, and chronic stroke patients with reduced function of the upper extremity. Patterns of CMI were explored to evaluate overall attentional capacity and attention allocation.

Our primary hypothesis was that CMI would be greater in both PD and stroke patients compared to age‐matched controls due to increased cognitive involvement in motor control, reduced attentional capacity, and/or deficits in attention allocation. We also hypothesized that a higher motor‐task complexity (i.e., catching targets while avoiding obstacles, compared to catching targets only) would have a detrimental effect on dual‐task performance within each group. It was anticipated that CMI would be greater in more severely affected patients, and that attention allocation would be a reflection of their cognitive and/or motor abilities.

## 2 MATERIALS AND METHODS

### 2.1 Participants

For this cross‐sectional study we recruited 57 patients with PD fulfilling the UK PD Brain Bank criteria (Gibb & Lees, 1988) and 57 chronic stroke patients (>8 weeks poststroke) with reduced function of the upper extremity as determined by the Fugl‐Meyer Upper Extremity Scale (FM‐UE; Fugl‐Meyer, Jääskö, Leyman, Olsson, & Steglind, 1975; see Table 1 for patient characteristics). Patients were recruited from the outpatient clinics of the Department of Neurology and the Department of Rehabilitation Medicine of the Leiden University Medical Center and from a list of patients who were discharged from the Rijnlands Rehabilitation Center between January 2013 and June 2014. Patients were excluded if they had disorders of the central nervous system or other conditions that could affect motor function of the upper extremity supplementary to PD or stroke. All patients were allowed to take their routine medications at the time of the experiment. Fifty‐seven healthy controls (23 women, 34 men; mean ± *SD* age: 63.8 ± 7.6 years), who were sex‐matched and age‐matched (±3 years) at group level to the patients, were recruited both through advertisements and from a database of volunteers who had participated in previous studies. Controls had normal or corrected to normal vision and hearing, had no apparent cognitive disorders or deficits, and had no history of disorders affecting the function of the upper extremities. Written informed consent was obtained according to the Declaration of Helsinki. The ethical committee of the Leiden University Medical Center approved the study protocol.

**Table 1:** Clinical characteristics of PD patients and stroke patients

	PD patients	Stroke patients
*N*	57	57
Sex (male/female)	36/21	33/24
Age (year; mean, *SD*)^a^	65.7 ± 8.9	61.4 ± 10.3
Disease duration (year; median, IQR)	11.8 [7.9–16.3]	3.8 [2.3–7.3]
Tested side (dominant/nondominant)	31/26	28/29
Reachable workspace area (m^2^; mean, *SD*)^b^	1.01 ± 0.15	0.79 ± 0.31*
PD‐specific clinical characteristics		
Hoehn and Yahr (median, range)^c^	3 [1–5]	–
Stereotactic surgery (yes/no)	6/51	–
MDS‐UPDRS‐III (mean, *SD*)^d^	36.6 ± 16.3	–
SCOPA‐COG (mean, *SD*)^e^	27.6 ± 7.0	–
Stroke‐specific clinical characteristics		
First ever stroke (%)	–	86
Type of stroke (ischemic/hemorrhage)	–	44/13
Lesion side (left/right/both)	–	32/22/3
Bamford classification^f^		
TACS (*n*)	–	6
PACS/POCS (*n*)	–	39
LACS (*n*)	–	9
FM‐UE (median, IQR)^g^	–	57 [20.5–62]
MoCA (median, IQR)^h^	–	25 [23–27]

^a^Not significantly different between PD patients and controls (*t*_112_ = −0.86, *p *=* *0.39) or between stroke patients and controls (*t*_112_ = 1.71, *p *=* *0.09). ^b^Reachable workspace area = product of the horizontal and vertical movement range of the wrist relative to the shoulder; ^c^0–5; high: worse; ^d^MDS‐UPDRS‐III, Movement Disorders Society sponsored revision of the Unified Parkinson's Disease Rating Scale, part III (motor evaluation); 0–132; high: worse; ^e^SCOPA‐COG, SCales for Outcomes in PArkinson's disease‐COGnition; 0–43; high: better. ^f^information available for 54 patients; TACS, Total anterior circulation stroke, PACS/POCS, Partial anterior/posterior circulation stroke; LACS, Lacunar stroke; ^g^FM‐UE, Fugl‐Meyer Upper Extremity Scale; 0–66; high: better; hMoCA, Montreal Cognitive Assessment; 0–30; high: better.

* Significantly reduced compared to controls (1.07 ± 0.15 m^2^, *p *<* *0.001).

### 2.2 Measurement instruments and data collection procedure

#### 2.2.1 Clinical assessment

Cognitive function was evaluated in PD patients using the SCales for Outcomes in PArkinson's disease‐COGnition (SCOPA‐COG; Marinus et al., 2003) and in stroke patients using the Montreal Cognitive Assessment (MoCA; Nasreddine et al., 2005). The severity of motor symptoms in PD patients was measured using the Hoehn and Yahr scale (Hoehn & Yahr, 1967) and section III of the Movement Disorder Society version of the Unified Parkinson's Disease Rating Scale (MDS‐UPDRS‐III; Goetz et al., 2008). The severity of upper‐limb motor symptoms in stroke patients was measured using the FM‐UE (Fugl‐Meyer et al., 1975). In controls, hand dominance was assessed using a Dutch version of the Edinburgh Handedness Questionnaire (Oldfield, 1971).

#### 2.2.2 Cognitive task

The auditory Stroop task (Cohen & Martin, 1975) was used as cognitive task. The words “high” and “low”, spoken by a woman's voice in either a high pitch or a low pitch, were presented to the participants with an interstimulus interval of 2 s. Participants were instructed to verbally indicate the pitch of the word they heard (ignoring the actual word presented) by responding “high” or “low” as accurately and as quickly as possible. Participants were allowed to correct their response before the next stimulus occurred. The stimuli (50% congruent and 50% incongruent, ordered randomly) were presented via a headset (Trust 15480 Comfortfit) and were recorded together with the responses using Moo0 Voice Recorder (version 1.4.3. http://www.moo0.com). The single‐task cognitive condition consisted of 11 stimuli (total duration: 30 s). During the dual‐task conditions, duration of the cognitive task was equal to that of the motor task (i.e., from start to finish of the motor task).

#### 2.2.3 Motor task

Participants sat in a chair or in their own wheelchair placed circa 1.5 m in front of a 60” LED TV (Sharp LC‐60LE652E, Sharp Electronics Europe Ltd., Usbridge, UK). Movements of the arms and trunk were recorded using a Microsoft Kinect^™^ v2 sensor that was mounted above the LED TV. Based on depth data obtained with an infrared laser transmitter and an infrared camera, the Kinect for Windows software development kit (SDK 2.0, http://www.microsoft.com) provided real‐time 3D‐coordinates of the wrist, elbow, shoulder, head, and trunk at a sampling rate of 30 Hz. D‐flow software (Motekforce Link, Amsterdam, The Netherlands; Geijtenbeek, Steenbrink, Otten, & Even‐Zohar, 2011) expanded with a data fusion component (NCF, Noldus, Wageningen, The Netherlands) was used for controlling the experiment and data storage.

**Figure 1:**
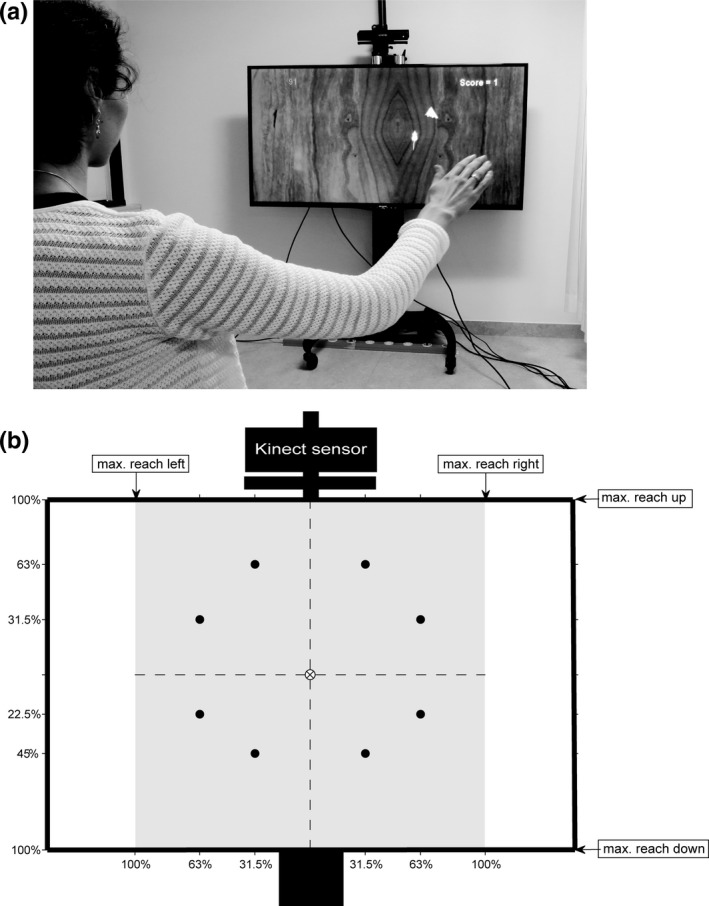
(a) Overview of the experimental setup; (b) Schematic representation of distribution of targets and obstacles (•) over the individually determined reachable workspace area. The center of the LED TV corresponded to the center of the participant's reachable workspace area (⊗). All positions of the virtual objects were scaled such that the upper and lower edges of the LED TV corresponded to the extremes of the participant's reachable workspace area. Targets were presented one at a time in a pseudorandom order. In the high‐difficulty conditions one‐third of the targets suddenly changed into an obstacle and the target appeared at a nearby location within the same quadrant

Participants performed unsupported goal‐directed movements in the frontal plane (Figure 1a) to control the horizontal and vertical movements of a virtual gray mouse, presented against a background of virtual wood on the LED TV, to collect virtual pieces of yellow cheese (targets) as fast as possible while avoiding a virtual black‐and‐white cat (obstacle; present in the high‐difficulty level only). Patients performed the task with their (most) affected arm. Controls were randomly assigned to perform the task with either their dominant arm (*n *=* *29) or nondominant arm (*n *=* *28).

Each condition of the motor task consisted of two series of 24 targets. Targets were evenly distributed over eight positions within the individually determined reachable workspace area (see Figure 1b) and were presented one at a time in a pseudorandom order (i.e., three blocks of eight targets; each target position was presented once within a block, in random order, to ensure that the eight target positions were evenly distributed within each condition; two targets within the same quadrant were always separated by at least one target in a different quadrant; there was no extra pause between these blocks of eight trials). The center of the LED TV corresponded to the center of the participant's reachable workspace area and all positions and movements of the virtual objects were scaled such that the upper and lower edges of the LED TV corresponded to the extremes of the participant's reachable workspace area. Hence, for all participants the targets were presented at the exact same positions on the LED TV, but the associated movement distance depended on the individually determined reachable workspace area. The horizontal and vertical positions of the virtual mouse on the LED TV were determined by the measured horizontal and vertical position of the wrist in the frontal plane (relative to the center of the participant's reachable workspace area). A first‐order filter (*τ* = 0.05 s) was applied to the wrist position signal to minimize the visual effects of high‐frequency measurement noise.

Prior to the start of each series of 24 targets, the participant moved the virtual mouse toward a virtual start button in the center of the LED TV. After a 5‐s countdown the first target appeared and the start button disappeared. A target was considered “caught” if the center of the virtual mouse was within 0.02 m from the center of the virtual cheese for 0.1 s. As soon as a target was caught, or if a target was not caught within 5 s after appearance, the target disappeared and the next target appeared. The participant thus moved the virtual mouse from one target to the next without returning to a “home position” in between.

To evaluate whether a higher complexity of the motor task would affect dual‐task performance, two difficulty levels of the motor task were introduced: catching targets (i.e., without obstacles; “low difficulty”) and catching targets while avoiding obstacles (“high difficulty”). In the high‐difficulty conditions, eight out of the 24 targets per series (i.e., 16 out of 48 targets per condition) suddenly changed into an obstacle and the target appeared at a nearby location within the same quadrant (see Figure 1b). The obstacle (i.e., a virtual cat) appeared as soon as the mouse was within a specific distance from the target (depending on movement velocity so that the time available for obstacle avoidance was circa 0.8 s for all participants). If an obstacle was hit, that is, if the center of the virtual mouse was within 0.03 m from the center of the virtual cat, both the obstacle and target disappeared and the next target appeared. The obstacles were presented in a pseudorandom order (i.e., once for each of the target positions (Figure 1b); evenly distributed between the first and second half of each series; two obstacles were always separated by at least one target without obstacle). Events in the motor task (e.g., start, appearance of target/obstacle, catch) were never accompanied by sound to avoid interference with the cognitive task under dual‐task conditions.

#### 2.2.4 Procedure

Participants performed the following conditions: (a) single cognitive task; (b) single low‐difficulty motor task, that is, without obstacles; (c) single high‐difficulty motor task, that is, with obstacles; (d) dual task: cognitive task and low‐difficulty motor task simultaneously; and (e) dual task: cognitive task and high‐difficulty motor task simultaneously. During dual‐task conditions, participants were instructed to perform both tasks to their best ability.

Prior to each of the five conditions, participants performed a short practice (four targets). The order of single‐task versus dual‐task conditions as well as the order of low‐ versus high‐difficulty levels within the motor task were randomized across participants. Patients who experienced limited physical capacity or complained of fatigue (four PD patients, 10 stroke patients) performed only one series per condition (i.e., 24 instead of 48 targets) to reduce the risk that not all conditions could be completed. After completing all conditions, participants rated the perceived “fun” and “difficulty” of the cognitive and motor task on 11‐point numeric rating scales (0: none, 10: maximum possible).

A subgroup of 12 PD patients, 12 stroke patients, and 12 healthy controls repeated the test after 1 week at the same hour of the day in order to determine test–retest reliability. Methodological details and results of this analysis are presented in Supporting Information Appendix S1A.

### 2.3 Data processing

Data was processed using MATLAB (The Mathworks Inc., Natick MA, USA, version R2016a). Performance on the cognitive task (*P*_C_ *,* in %s^−1^) was calculated as the percentage of correct answers (determined from the sound recordings) divided by the average response time of correct responses (determined from the sound recordings using a custom‐made algorithm). Performance on the motor task (*P*_M_ *,* in %s^−1^) was calculated as the percentage of collected targets divided by the average “catch time” (i.e., time in seconds between target appearance and catch).

Dual‐task effect (*DTE*) was calculated as:

(1) $$\text{DTE}\left(\%\right)=\frac{\text{dual‐task performance ‐ single‐task performance}}{\text{single‐task performance}}\times 100\%$$

separately for the cognitive task (*DTE*_C_) and motor task (*DTE*_M_), and separately for conditions involving the low‐difficulty and high‐difficulty motor task. Negative *DTE* values indicate performance deterioration, or dual‐task cost, while positive *DTE* values indicate an improvement, or dual‐task benefit (Plummer & Eskes, 2015). *DTE*_total_ was calculated as the average of *DTE*_C_ and *DTE*_M_ to provide an overall index of CMI. *Priority* was calculated as *DTE*_M_ – *DTE*_C_, with positive values indicating motor priority and negative values indicating cognitive priority.

**Figure 2:**
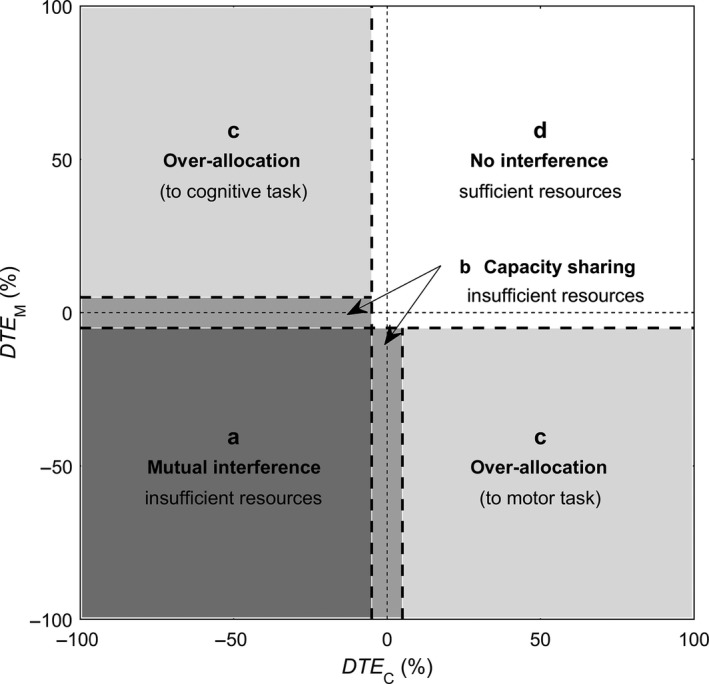
Patterns of CMI (based on Plummer et al., 2013, 2014; Plummer & Eskes, 2015): (a) both tasks deteriorate (“mutual interference”), indicating insufficient attentional resources; (b) deteriorated performance on one of the tasks but not the other (“capacity sharing with primary allocation to one task”), indicating that one of the tasks is prioritized in an attempt to preserve performance in this domain when attentional resources are insufficient; (c) improvement on one task at the cost of deteriorated performance on the other task (“over‐allocation of attention to one task”), which may be but not necessarily is due to insufficient attentional resources; (d) no interference or even facilitation, indicating sufficient attentional resources. Dotted lines at −5% and +5% indicate threshold values for interference and facilitation

Based on the values of *DTE*_C_ and *DTE*_M_, participants were classified according to the following patterns of CMI (see Figure 2, based on Plummer et al., 2013, 2014; Plummer & Eskes, 2015): (a) mutual interference, insufficient attentional resources; (b) capacity sharing with primary allocation to one task, insufficient attentional resources; (c) over‐allocation of attention to one task; (d) no interference, sufficient attentional resources. Threshold values for interference and facilitation were set at −5% and +5%.

### 2.4 Statistical analysis

In total 49 PD patients, 45 stroke patients and 56 controls (i.e., 150 out of the 171 originally included participants) were included in the group comparisons and analysis of CMI patterns. Participants were excluded from all statistical analyses for various reasons. Five PD patients and one stroke patient were unable to complete one or more tasks due to fatigue (caused by the larger study protocol where this experiment was part of). *P*_C_ could not be evaluated in one PD patient with severe speech problems and in another PD patient due to technical issues. One control participant was unable to perform the cognitive task. *P*_M_ could not be evaluated in 11 stroke patients who had a very limited reachable workspace area (<0.2 m^2^). *DTE*_M_ could not be calculated in one PD patient due to a single‐task *P*_M_ of 0%s^−1^.

All statistical analyses were performed using IBM^®^ SPSS^®^ Statistics 23.0 (IBM Corp., Armonk NY). Normality curves were inspected and Kolmogorov–Smirnov tests were used to assess whether data were normally distributed. In total six outliers were observed for *DTE*, which were attributable to very low baseline values (distributed over one PD patient, three stroke patients, and one control participant; equally distributed over cognitive/motor tasks and low‐/high‐difficulty levels). To prevent these outliers from having a disproportionate impact on the statistical analysis of this variable, they were replaced by the mean minus two standard deviations of the remainder of the group (Field, 2009).

Statistical analyses were conducted to compare either PD patients versus controls and stroke patients versus controls. Group differences in single‐task performance in both the cognitive and motor domain were first evaluated. Single‐task *P*_C_ was compared between groups (PD patients vs. controls, stroke patients vs. controls) using independent *t* tests. Single‐task *P*_M_ was submitted to mixed analyses of variance (ANOVAs) with group (separate analyses for comparing PD vs. control, or stroke vs. control) as between‐subject factor and difficulty (low vs. high) as within‐subject factor. To test our hypotheses that CMI would be greater in patients compared to controls, and that a higher complexity of the motor task would be detrimental to dual‐task performance, *DTE* was submitted to mixed ANOVAs with group (PD vs. control or stroke vs. control) as between‐subject factor and with task (cognitive vs. motor) and motor‐task difficulty (low vs. high) as within‐subject factors. In order to explore whether *DTE* results were influenced by single‐task performance, which is in the denominator of Equation (1), we repeated the analysis of *DTE* using a linear mixed model with single‐task performance as a covariate. In a similar way, we explored whether single‐task *P*_M_ and *DTE* results were influenced by the individually determined reachable workspace area (results are presented in Supporting Information Appendix S1B). Effect sizes were quantified as Pearson's *r* for independent *t* tests and as partial eta squared ($\eta_{p}^{2}$ ) for ANOVAs. Significance was set at *p *<* *0.05. For ANOVAs, significant interaction effects were analyzed using simple effects analyses, which yielded the effect of one independent variable at individual levels of the other independent variable (Field, 2009).

To explore whether patterns of CMI differed between patients and controls, we used chi‐square tests to compare the overall frequency distribution of CMI patterns for PD patients versus controls and for stroke patients versus controls, separately for each difficulty level of the motor task. Effect size was quantified as Kramer's *V*.

Within each patient group, we aimed to determine whether CMI was greater in more severely affected patients. To this end, we first calculated a “combined clinical severity score” (CCSS) for each patient from the clinical ratings of cognitive function and motor function. Clinical ratings were converted to *Z*‐scores (for PD patients) or rankings (for stroke patients) and averaged over the two domains, such that lower CCSS values reflected more severely affected patients. It was evaluated whether CCSS was associated with overall dual‐task interference (*DTE*_total_) using Pearson's correlation coefficient for PD patients and Spearman's correlation coefficient for stroke patients. We subsequently evaluated whether attention allocation reflected the cognitive and/or motor abilities. Partial correlation analyses were used within each patient group to assess the unique contribution of impairments in the cognitive domain (correcting for clinical ratings of motor function) and impairments in the motor domain (correcting for clinical ratings of cognitive function) to dual‐task effects (*DTE*_total_, *DTE*_C_ *,* and *DTE*_M_) and *Priority*. In specific, SCOPA‐COG score and MDS‐UPDRS‐III score were used within the PD group as clinical ratings of cognitive function and motor function, respectively, and Pearson's correlation coefficient was used for partial correlations. MoCA score and FM‐UE score were used within the stroke group as clinical ratings of cognitive function and motor function, respectively, and Spearman's correlation coefficient was used for partial correlations. Within each group, we also explored whether attention allocation was related to perceived “fun” and “difficulty” of the tasks (methodological details and results of this analysis are presented in Supporting Information Appendix S1C).

## 3 RESULTS

Significant results for group comparisons of single‐task performance, dual‐task effects, and patterns of CMI are presented in Table 2. Results of associated posthoc analyses are described in the following sections. Correlation coefficients between dual‐task effects and clinical tests are presented in Table 3.

**Table 2:** Significant statistical results for group comparisons of single‐ and dual‐task performance and patterns of CMI

Outcome	Effect	PD versus controls				Stroke versus controls			
		Test statistic		*p*	Effect size	Test statistic		*p*	Effect size
Single‐task performance									
*P*_C_ a	G	—	—			*t*_99_ =	4.40	<0.001	0.40
*P*_M_ b	G	*F*_1,103_ =	41.61	<0.001	0.29	*F*_1,99_ =	41.98	<0.001	0.30
	D	*F*_1,103_ =	290.62	<0.001	0.74	*F*_1,99 =_	221.14	<0.001	0.69
	G × D	*F*_1,103_ =	12.05	0.001	0.11	*F*_1,99_ =	23.09	<0.001	0.19
Dual‐task effects									
*DTE* b	G	*F*_1,103_ =	15.40	<0.001	0.13	—	—		
	T	*F*_1,103_ =	8.39	0.005	0.08	—	—		
	D	*F*_1,103_ =	15.28	<0.001	0.13	*F*_1,99_ =	16.31	<0.001	0.14
	T × D	*F*_1,103_ =	95.00	<0.001	0.48	*F*_1,99_	45.12	<0.001	0.31
Patterns of CMI (frequency distribution) c									
Low‐difficulty	G	*χ*_1,3_ =	16.44	<0.001	0.40	—	—		
High‐difficulty	G	*χ*_1,3_ =	7.12	0.07	0.26	*χ*_1,3_ =	8.02	0.04	0.28

Comparisons were based on *n *=* *56 controls versus *n *=* *54 PD patients, and on *n *=* *56 controls versus *n *=* *45 stroke patients. G, group, as indicated; D, motor‐task difficulty (low vs. high, for *P*_M_ and *DTE*); T, task (cognitive vs. motor, for *DTE* only).

^a^Independent *t* tests, effect size quantified as Pearson's *r*; ^b^Mixed ANOVAs, effect size quantified as partial eta squared ($\eta_{p}^{2}$ ); ^c^Chi‐squared tests, effect size quantified as Kramer's *V*.

**Table 3:** Correlation with clinical tests of cognitive and motor function

Difficulty	PD						Stroke					
	CCSS^a^		SCOPA‐COG b		MDS‐UPDRS‐III^c^		CCSS^a^		MoCA^d^		FM‐UE^e^	
	Low	High	Low	High	Low	High	Low	High	Low	High	Low	High
*DTE*_total_	0.29*	0.34*	0.24	0.18	−0.05	−0.17	0.38*	0.24	0.08	0.06	0.32*	0.22
*DTE*_C_	–	–	0.20	0.29*	0.00	−0.14	–	–	0.01	0.10	0.29	0.05
*DTE*_M_	–	–	0.23	0.02	−0.08	−0.17	–	–	0.06	0.00	0.15	0.23
*Priority*	–	–	0.06	−0.21	−0.10	−0.02	–	–	−0.05	−0.13	0.00	0.17

(Partial) correlations were calculated using Pearson's correlation coefficient for PD patients and Spearman's correlation coefficient for stroke patients.

^a^CCSS, combined clinical severity score, calculated for each patient from the clinical ratings of cognitive function and motor function. ^b^controlled for MDS‐UPDRS‐III; ^c^controlled for SCOPA‐COG; ^d^controlled for FM‐UE; ^e^controlled for MoCA.

* *p *<* *0.05. For CCSS, SCOPA‐COG, MoCA, and FM‐UE higher scores indicate better function, whereas for MDS‐UPDRS‐III lower scores indicate better function. Negative values of DTE_total_, DTE_C_ *,* and DTE_M_ indicate cognitive‐motor interference. Negative values of *Priority* indicate prioritization of the motor task over the cognitive task.

### 3.1 Single‐task performance

**Figure 3:**
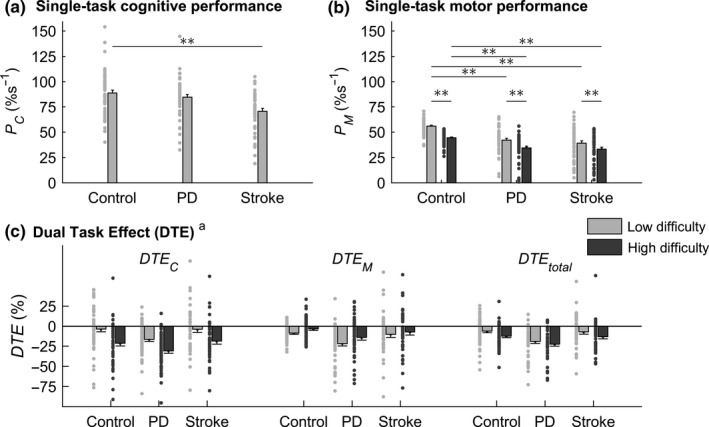
Results for (a) single‐task cognitive performance *P*_C_; (b) single‐task motor performance *P*_M_; (c) dual‐task effects in each domain (cognitive: *DTE*_C_; motor: *DTE*_M_) complemented by the overall dual‐task effect (*DTE*_total_). Individual data points are presented. Bars represent mean values and error bars represent standard errors. **p <* *0.01. ^a^ Statistical results for *DTE* are presented in Table 2 and described in the text

Single‐task *P*_C_ was not significantly different between PD patients and controls (Figure 3a), whereas single‐task *P*_M_ was significantly lower in PD patients compared to controls (Figure 3b). Both single‐task *P*_C_ and single‐task *P*_M_ were lower in stroke patients compared to controls (Figure 3a,b). In all three groups, *P*_M_ was lower for the high‐difficulty compared to the low‐difficulty level of the motor task (*p *<* *0.001). This difficulty effect was more pronounced for controls ($\eta_{p}^{2}$ = 0.69) than for PD patients ($\eta_{p}^{2}$ = 0.46) and stroke patients ($\eta_{p}^{2}$ = 0.32).

### 3.2 Dual‐task effects

Parkinson's disease patients experienced more interference (i.e., more negative values of *DTE*) than controls (main effect of group; Figure 3c). *DTE* was not different between stroke patients and controls (main effect of group, *p *=* *0.81; Figure 3c). There were no significant interactions between group and task or motor‐task difficulty. Follow‐up analyses on the interaction between task and motor‐task difficulty yielded largely similar results for analyses based on PD/controls and analyses based on stroke/controls. In specific, interference on the cognitive task markedly increased for the high‐difficulty compared to the low‐difficulty level of the motor task (i.e., more negative *DTE*_C_ when obstacles were introduced; PD/controls: *p *<* *0.001, $\eta_{p}^{2}$ = 0.45; stroke/controls: *p *<* *0.001, $\eta_{p}^{2}$ = 0.35) while interference on the motor task tended to decrease (i.e., slightly less negative *DTE*_M_; PD/controls: *p < *0.001, $\eta_{p}^{2}$ = 0.13; *s*troke/controls: *p *=* *0.05, $\eta_{p}^{2}$ = 0.04). The high‐difficulty motor task was prioritized over the cognitive task (i.e., *DTE*_C_ more negative than *DTE*_M_; PD/controls: *p *<* *0.001, $\eta_{p}^{2}$ = 0.29; stroke/controls: *p *<* *0.001, $\eta_{p}^{2}$ = 0.19), whereas the cognitive task tended to be prioritized over the low‐difficulty motor task (i.e., *DTE*_C_ less negative than *DTE*_M_ *;* PD/controls: *p *=* *0.02, $\eta_{p}^{2}$ = 0.06; stroke/controls: *p *=* *0.06, $\eta_{p}^{2}$ = 0.04).

### 3.3 Patterns of CMI

**Figure 4:**
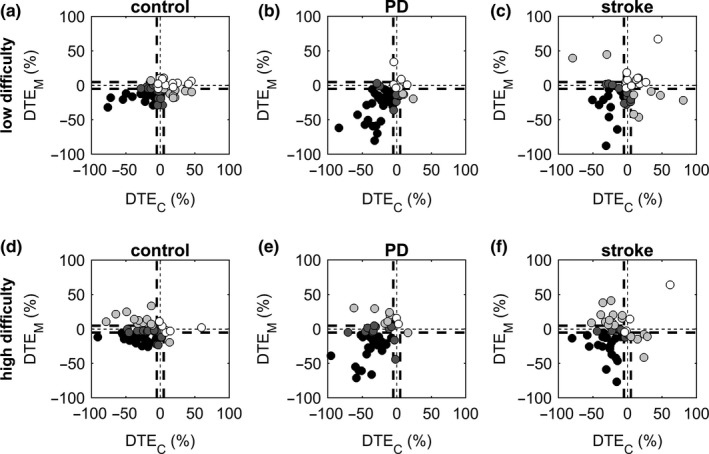
Patterns of CMI for controls (a, d), PD patients (b, e) and stroke patients (c, f), with separate plots for the low‐difficulty (a–c) and high‐difficulty (d–f) level of the motor task. Each circle represents one patient. Based on values of *DTE*_C_ and *DTE*_M_, circles are color‐coded according to the four main patterns of CMI presented in Figure 2: black = mutual interference; dark gray = capacity sharing with primary allocation to one task; light gray = overallocation of attention to one task; white = no interference, or facilitation. Dotted lines at −5% and +5% indicate threshold values for interference and facilitation

The frequency distribution of participants over the four patterns of CMI was significantly different between PD patients and controls for the low‐difficulty level of the motor task ($\chi_{3}^{1}$ = 16.44, *p* < 0.001, *V* = 0.40). This difference can easily be appreciated from Figure 4a,b: 67% of the PD patients fell within the “mutual interference” category (i.e., the lower left quadrant), compared to only 29% of controls. This difference between PD patients and controls failed to reach significance for the high‐difficulty level of the motor task ($\chi_{3}^{1}$ = 7.12, *p *=* *0.07, *V *=* *0.26).

The frequency distribution of participants over the four patterns of CMI was similar between stroke patients and controls for the low‐difficulty level of the motor task ($\chi_{3}^{1}$ = 0.37, *p *=* *0.96, *V *=* *0.06), but differed between these groups for the high‐difficulty level of the motor task ($\chi_{3}^{1}$ = 8.02, *p *=* *0.04, *V *=* *0.28). From Figure 4d,f, it can be appreciated that stroke patients more often fell within the “mutual interference” category (42% of stroke patients vs. 32% of controls) or the “over‐allocation” category (38% of stroke patients vs. 23% of controls), while controls more often fell within the “capacity sharing” category (11% of stroke patients vs. 34% of controls).

### 3.4 Correlations with clinical tests

Significant positive correlations were observed between CCSS and *DTE*_total_ within both patient groups (Table 3). More severely affected patients (i.e., patients with more negative values of CCSS) thus experienced more CMI under dual‐task conditions (reflected by more negative values of *DTE*_total_) than less affected patients.

Associations with impairments in either domain (cognitive, motor) can also be appreciated from Table 3. For PD patients, reduced cognitive function (i.e., lower score on SCOPA‐COG) was associated with more deterioration of the cognitive task under dual‐task conditions (i.e., more negative *DTE*_C_). Impaired motor function (i.e., higher score on the MDS‐UPDRS‐III) was not associated with any DTE measure. For stroke patients, reduced cognitive function (i.e., lower score on MoCA) was not associated with any DTE measure, whereas impaired motor function (i.e., lower score on the FM‐UE) was associated with more dual‐task interference (i.e., more negative *DTE*_total_). For both groups, no significant associations were observed between *Priority* and clinical ratings of cognitive or motor function.

## 4 DISCUSSION

To our knowledge, this is the first study that systematically evaluated patterns of CMI during upper‐limb motor control in a large sample of healthy individuals and two highly prevalent neurological conditions associated with deficits of cognitive and motor processing (PD and stroke).

As expected, healthy individuals experienced CMI during simultaneous performance of a cognitive task and a goal‐directed upper‐limb motor task, especially under challenging high‐difficulty conditions of the motor task. Interference on the cognitive task markedly increased when obstacles were introduced in the motor task (i.e., more negative values of *DTE*_C_), whereas interference on the motor task slightly decreased (i.e., less negative values of *DTE*_M_). The high‐difficulty motor task thus demanded—and was allocated—more attention than the low‐difficulty motor task, albeit at the cost of a deterioration of cognitive task performance (illustrated in Figure 4 by a shift toward the left side in all groups, most clearly observed in the control group). The low‐difficulty motor task was associated with less interference on the cognitive task, without a clear prioritization of one of the tasks (at group level).

In accordance with our hypotheses, patients with neurological deficits showed different patterns of CMI compared to healthy individuals, depending on diagnosis (PD or stroke) and severity of cognitive and/or motor symptoms. PD patients experienced greater CMI than controls, with the majority of patients showing interference in both the cognitive and the motor domain. Attentional demand thus exceeded capacity (i.e., attentional resources were insufficient) in the majority of PD patients. In contrast to our expectations, stroke patients in general did not experience greater CMI than controls. Substantial heterogeneity within this patient group (in terms of lesion location and severity of cognitive and motor impairments) may have played a role in this regard. Indeed, the patterns of CMI were more variable within the group of stroke patients compared to the control group, especially with regard to *DTE*_M_ (i.e., larger dispersion along the *y*‐axis of Figure 4).

In both patient groups the correlation between CCSS and *DTE*_total_ indicated that CMI was greater in more severely affected patients. Differences between the two patient groups become apparent concerning the unique contributions of impairments in the cognitive and motor domain. Within the group of PD patients, the degree of interference during dual‐task conditions appeared more related to cognitive function than to motor function. This finding potentially illustrates the impact of cognitive impairments on daily life activities in PD patients (Leroi, McDonald, Pantula, & Harbishettar, 2012; Rosenthal et al., 2010), who depend on cortical executive control even for routine tasks due to basal ganglia dysfunction (Redgrave et al., 2010). In contrast, within the group of stroke patients the degree of interference during dual‐task conditions appeared to be more related to motor function than to cognitive function. This suggests that especially stroke patients with severe motor dysfunction experience CMI due to increased cognitive involvement in motor control (in line with Houwink et al., 2013). Although circa 50% of the included stroke patients fulfilled the criteria for mild cognitive impairment (i.e., MoCA score <26), the impact of these relatively mild cognitive symptoms seems limited.

Previous studies, which have mainly focused on CMI during highly automated gross motor activities such as walking or maintaining balance, have revealed that healthy individuals typically show a reduction of walking speed or an increase of variability measures (indicating reduced stability) while dual‐tasking. Stronger effects have been reported in elderly subjects, in subjects with mild cognitive impairment, in PD patients, and in subacute and chronic stroke patients with globally intact cognition (for reviews see Kelly et al., 2012; Amboni et al., 2013; Plummer et al., 2013). Although no definitive strategy regarding attention allocation and task prioritization has been identified, it should be noted that most studies reported interference in gait or balance, while DTEs in the cognitive domain were more variable (Plummer et al., 2013; Rochester et al., 2014; Smulders, van Swigchem, de Swart, Geurts, & Weerdesteyn, 2012). Similar findings of interference in the motor domain (with variable DTEs in the cognitive domain) have been reported in a few small studies involving upper‐limb motor tasks such as writing (Broeder et al., 2014), circle drawing (Houwink et al., 2013), and isometric force matching (Alberts et al., 2008; Frankemolle et al., 2010). In line with these previous studies we also observed interference in the motor domain, which was accompanied by interference in the cognitive domain to a greater or lesser extent depending on task difficulty. Our results suggest that the motor task was allocated more attention when its difficulty was increased, at the expense of increased interference in the cognitive domain. The large variation in patterns of CMI within each group (Figure 4), however, points to considerable interindividual differences in attentional capacity and attention allocation.

Our results further suggest that healthy individuals were flexible in their attention allocation (see also Supporting Information Appendix S1C): they tended to prioritize the more “fun” task when task complexity allowed (i.e., with low‐difficulty motor task), whereas they prioritized the motor task under more challenging conditions (i.e., with obstacles, high‐difficulty motor task), perhaps to preserve at least a “minimally acceptable level of performance”. Patients with neurological deficits seemed less flexible in their strategy: performance in dual‐task conditions appeared more related to their cognitive and/or motor abilities than to fun ratings for the respective tasks. Attention allocation, however, was not simply reflective of cognitive or motor abilities. Together, these findings underscore that the mediators of dual‐task interference are more complex than cognitive and motor abilities combined with “a core motivation to minimize danger and maximize pleasure” (Williams, 2006): also the cognitive reserve, compensatory abilities, personality, affect and expertise may play a role (Yogev‐Seligmann, Rotem‐Galili, Dickstein, Giladi, & Hausdorff, 2012). The self‐selected strategy for task prioritization may thus differ between individuals, between different combinations of dual‐tasks (e.g., when difficulty of the motor task is increased), and even between measurement sessions (which may result in low test–retest reliability for DTE measures, see Supporting Information Appendix S1A).

Compared to previous works, our study has some important advantages. Firstly, our study includes a large(r) number of participants with a varying degree of cognitive and motor impairments, which allowed us to not only compare CMI between patients and controls, but also to explore the associations between DTEs and clinical tests of cognitive and motor function. Secondly, our study provides insight into the relationship between DTEs in the cognitive and motor domain (on group level as well as on individual level), revealing different patterns of CMI between groups and between individuals. Thirdly, speed–accuracy trade‐off is taken into account in quantifying cognitive performance. A limitation of this study is that the upper‐limb motor task could not be performed in severely affected patients with a very limited reachable workspace area (<0.2 m^2^) because measurement errors were relatively large compared to the small amounts of voluntary movement, especially when the arm was held close to the trunk (as is often the case in severely affected stroke patients). This may have biased our results toward an underestimation of CMI in stroke patients. Before drawing general conclusions from this study, several other considerations should be taken into account as well. Firstly, our study was not intended to find the specific brain areas involved in CMI. This would require a more homogenous stroke population in terms of location of the lesion. Secondly, additional analyses presented in Supporting Information Appendix S1B showed that our findings, which were obtained in patients with reachable workspace >0.2 m^2^, were not attributable to or distorted by individual differences in reachable workspace area (and the associated differences in movement distance between the targets) or individual differences in single‐task performance. When evaluating CMI in individual patients, however, it should be taken into account that a small deterioration or improvement of performance under dual‐task conditions can lead to disproportionally large *DTE* values in patients with low single‐task performance. For example, the greater variation of CMI patterns within the group of stroke patients (Figure 4) is partly due to low single‐task performance in the cognitive and/or motor domain. Changes in absolute measures of single‐ and dual‐task performance (e.g., dual‐task *P*_M_ in %s^−1^) should therefore be considered in addition to relative measures of dual‐task performance (*DTE* in %; as recommended by Agmon, Kelly, Logsdon, Nguyen, & Belza, 2015; Plummer & Eskes, 2015). Thirdly, the DTE measures in this cross‐sectional study provided useful insight into processes underlying CMI: they were sensitive to different levels of task complexity, different neurologic conditions, and different levels of disease severity. Unfortunately, test–retest reliability of the DTE measures appeared to be insufficient for use in longitudinal studies (see Supporting Information Appendix S1A). Fourthly, the present study focused on a gross measure of upper‐limb motor control (i.e., percentage of collected targets divided by the average “catch time”), but the collected motion data also allows for a more detailed analysis of motor function (e.g., quantifying the relative contribution of arm vs. trunk movements in stroke patients and evaluate changes in their relative contribution in response to treatment (van Kordelaar et al., 2012)). Finally, current time‐consuming steps in postprocessing (e.g., the manual scoring of responses on the cognitive test and manual removal of “non‐responses” that was required in some cases) need to be further automated for implementation in the clinical setting.

Within the patient groups only weak associations between clinical ratings of cognitive or motor function and DTE measures were observed, suggesting that DTE measures reflect a different construct than the unidimensional clinical tests. It remains to be investigated whether these DTE measures are a better indicator of difficulties with daily life activities that require adequate interaction with the environment and/or involve the simultaneous performance of two or more tasks. Our current findings underscore the added value of DTE measures in both the cognitive and motor domain, as they provide insight into overall attentional capacity as well as attention allocation in patients with neurological deficits. It may tentatively be suggested that dual‐task training (if possible using increasing levels of task complexity) provides opportunities for improving upper‐limb motor control in daily life.

In conclusion, our findings show that healthy individuals experienced CMI during simultaneous performance of a cognitive task and a goal‐directed upper‐limb motor task, especially under challenging conditions of the motor task. CMI was greater in PD patients, presumably due to insufficient attentional capacity in relation to increased cognitive involvement in motor control. Although no general increase of CMI was observed in chronic stroke patients, our results suggest that especially stroke patients with severe motor dysfunction experience CMI due to increased cognitive involvement in motor control.

## CONFLICT OF INTEREST

The authors have no competing interests.

## DATA ACCESSIBILITY

Supporting data are available from the DANS archive at https://doi.org/10.17026/dans-zjt-aehh.

## AUTHOR CONTRIBUTIONS

P.J.M. Bank: conception, organization and execution of research project; design and execution of analyses; writing of the first draft of the manuscript, critical revision of the manuscript. J. Marinus: conception and organization of research project; review and critique of statistical analysis and manuscript. R.M. van Tol: execution of research project; review and critique of manuscript. I.F. Groeneveld: assistance in recruitment of stroke patients; review and critique of manuscript. P.H. Goossens: assistance in preparation of research project, assistance in recruitment of stroke patients; review and critique of manuscript. J.H. de Groot: conception of research project; review and critique of statistical analysis and manuscript. J.J. van Hilten: conception of research project; review and critique of manuscript. C.G.M. Meskers: conception of research project; review and critique of statistical analysis and manuscript.
