# Supplementary material for: Cognitive‐motor interference during goal‐directed upper‐limb movements
Source: Eur J Neurosci. 2018 Oct 20;48(10):3146–58. doi: 10.1111/ejn.14168 (PMC6282826; doi:10.1111/ejn.14168)
Supplement: Supplementary file 2 [file EJN-48-3146-s001.pdf]

Supporting Information A ─ Assessment of test-retest reliability

It has been recommended to assess performance in both the cognitive and motor domain under single- and dual-task conditions to better evaluate changes in CMI in response to treatment (Plummer & Eskes, 2015). As a first step in evaluating whether the DTE measures (as presented in the main manuscript) can be used for longitudinal studies and for longitudinal monitoring of individual patients in clinical practice, we assessed the test-retest reliability of the DTE measures in a subgroup of 36 participants.

Methods

A subgroup of 36 participants, i.e., 12 PD patients, 12 stroke patients (see Table A.1) and 12 healthy controls (6 women, 6 men; mean ± SD age: 65.2 ± 6.8 years), repeated the test after one week at the same hour of the day in order to determine test-retest reliability. Participants rated the perceived change in function of the arm/hand on an 11-point numeric rating scale (0: much worse, 5: unchanged, 10: much better). The same order of tasks and conditions (single-task vs. dual-task, low vs. high difficulty) was kept between both measurements. The experimental procedure and data preprocessing were performed as presented in main manuscript.

Test-retest reliability of single-task performance (P_C and P_M), dual-task effects (DTE_{total}, DTE_C and DTE_M) and *Priority* was assessed by means of the intraclass correlation coefficient for absolute agreement ($ICC_{A,1}$) (de Vet *et al.*, 2006) based on all participants in this test-retest study (i.e. controls, PD patients and stroke patients combined). With data from 36 subjects, a ‘good’ ICC (> 0.6) would at least be detected as being ‘fair’ (> 0.4) (expected ICC = 0.61, 95% CI width = 0.42) (Shoukri *et al.*, 2004). $ICC_{A,1}$ values above 0.40 were considered fair, values above 0.60 were considered good, and values above 0.75 were

considered excellent (Cicchetti, 1994). $ICC_{A,1}$ values were complemented by mean differences and precision values obtained with a Bland-Altman analysis (i.e., the bias and limits of agreement) (Bland & Altman, 1999).

Results

11 PD patients, 11 stroke patients and 11 controls (i.e., 33 out of the 36 originally included participants) were included for analysis of test-retest reliability. One PD patient was excluded from the analyses because a single-task P_M of 0%s^{-1} precluded calculation of DTE_M, one stroke patient had a very limited reachable workspace area (< $0.2m^2$) that made evaluation of P_M impossible, and one control participant was not able to perform the cognitive task.

The self-reported function of the arm and hand was rated 5 (i.e. 'unchanged' as compared to the first measurement) by all controls, 10 out of 11 PD patients, and 8 out of 11 stroke patients participating in the test-retest study; one PD patient and one stroke patient reported a 1 (out of 5) point reduction; two stroke patients reported an improvement of 1 and 3 points, respectively.

Test-retest reliability was fair for single-task P_C and excellent for single-task P_M (Table A.2), with relatively small bias and narrow limits of agreement for these parameters. Test-retest reliability was fair for DTE_{total} and for DTE_M (high-difficulty level of the motor task). In contrast, test-retest reliability was poor for DTE_C, DTE_M (low-difficulty level of the motor task) and *Priority*, as was evidenced by $ICC_{A,1}$ values <.20 in combination with wide limits of agreement.

Table A.1. Clinical characteristics of PD patients and stroke patients in the test-retest study

	PD patients	stroke patients
N	12	12
Sex (male/female)	6 / 6	8/4
Age (yr) (mean, SD) [a]	69.9 ± 6.6	59.3 ± 10.5
Disease duration (yr) (median, IQR)	14.3 [8.4-21.1]	2.8 [2.2-22.1]
Tested side (dominant/non-dominant)	6/6	7/5
Reachable workspace area (m^2) (mean, SD) [b]	1.01 ± 0.16	0.72 ± 0.41 *
PD-specific clinical characteristics		
Hoehn and Yahr (median, range) [c]	3 [2-4]	–
Stereotactic surgery (yes/no)	0 / 12	–
MDS-UPDRS-III (mean, SD) [d]	39.1 ± 14.6	–
SCOPA-COG (mean, SD) [e]	26.7 ± 6.8	–
Stroke-specific clinical characteristics		
First ever stroke (%)	–	82
Type of stroke (ischemic/hemorrhage)	–	9/2
Lesion side (left/right/both)	–	7/3/1
Bamford classification [f]		
TACS (n)	–	0
PACS/POCS (n)	–	7
LACS (n)	–	0
FM-UE (median, IQR) [g]	–	56.5 [27-63]
MoCA (median, IQR) [h]	–	24.5 [22-26]

[a] Not significantly different between PD patients and controls (t_{22} = -1.73, P = .10) or between stroke patients and controls (t_{22} = 1.64, P = .11). [b] Reachable workspace area = product of the horizontal and vertical movement range of the wrist relative to the shoulder; * significantly reduced compared to controls (1.03 ± 0.17 m^2, P = .008). [c] 0-5; high: worse; [d] MDS-UPDRS-III = Movement Disorders Society sponsored revision of the Unified Parkinson’s Disease Rating Scale, part III (motor evaluation); 0-132; high: worse; [e] SCOPA-COG = SCales for Outcomes in PArkinson's disease-COGnition; 0-43; high: better. [f] TACS = Total anterior circulation stroke, PACS/POCS = Partial anterior/posterior circulation stroke; LACS = Lacunar stroke; [g] FM-UE = Fugl-Meyer Upper Extremity Scale; 0-66; high: better; [h] MoCA = Montreal Cognitive Assessment; 0-30; high: better.

Table A.2. Results

	Low motor-task difficulty			High motor-task difficulty		
	$ICC_{A,1}$	Bias [limits of agreement]		$ICC_{A,1}$	Bias [limits of agreement]	
Single-task P_C	.40	11.0	[3.3 18.6]	–	–	
Single-task P_M	.92	4.0	[2.2 5.8]	.84	4.3	[2.1 6.4]
DTE_{total}	.36	-1.3	[-7.5 4.8]	.29	-6.0	[-12.8 0.8]
DTE_C	.12	-1.4	[-11.6 8.9]	-.08	-11.2	[-21.9 -0.4]
DTE_M	.17	-1.3	[-11.2 8.6]	.55	-0.8	[-8.4 6.8]
Priority	-.07	0.2	[-15.9 16.0]	.18	10.4	[-2.4 23.1]

Discussion

Test-retest reliability was fair to excellent for single-task performance, with a small but positive bias indicating a learning effect. Test-retest reliability was poor to fair for DTE measures and *Priority,* with slightly less interference on the cognitive task (DTE_C) during the second measurement. Apart from these small learning effects, two possible reasons can be put forward for the low test-retest reliability of the DTE measures. Firstly, DTE measures were calculated from two to four parameters (single-task and dual-task performance on cognitive and/or motor task). Combining several individual outcome parameters, each with a potential measurement error, into a difference and ratio score (such as *DTE*) typically yields lower test-retest reliability as compared to individual outcome parameters. Secondly, participants may have applied different strategies during the two measurement sessions (e.g., prioritize motor task during one session, prioritize cognitive task during other session). Such change in strategy would result in low test-retest reliability of DTE_C and DTE_M. Since DTE_{total} reflects a combination of DTE in both the cognitive and motor domain, one might expect that DTE_{total} would be less susceptible to changes in strategy. However, a low $ICC_{A,1}$ was also observed for DTE_{total}, possibly because it was susceptible to measurement errors from four contributing parameters. Although participants may have applied a different strategy, it is reasonable to

expect that the underlying pattern of CMI (visualized in Fig. 2 of the main manuscript) would remain unchanged during the two measurement sessions. Exploratory analysis revealed that this was indeed the case for 42% of participants in the low-difficulty condition. Another 36% of participants were assigned to a “better” pattern of CMI during the second measurement (respectively 36% and 46% for the high-difficulty level of the motor task), which might be indicative of a learning effect.

In the cross-sectional study presented in the main manuscript, the DTE measures provided useful insight into processes underlying CMI: they were responsive to different levels of task complexity, different neurologic conditions and different levels of disease severity. Unfortunately the test-retest reliability of these DTE measures appears insufficient for use in longitudinal studies (e.g., evaluation of response to an intervention) or longitudinal monitoring of individual patients in clinical practice. It is recommended to increase practice time in order to minimize potential learning effects during the actual assessment. Repeated evaluation of CMI in individual patients, however, will still be complicated by potential changes in strategy.

Supporting Information B – Reachable workspace area or single-task performance as covariate

For all participants the virtual objects (targets and obstacles) of the motor task were presented at the exact same positions on the LED TV, but the associated movement distance depended on the individually determined reachable workspace area. In order to explore whether single-task motor performance (single-task P_M) results were influenced by reachable workspace area, we repeated the analysis of single-task P_M using ANCOVAs with workspace area as a covariate, with group as between subject factor (separate analyses for PD patients vs. controls and for stroke patients vs. controls) and with motor-task difficulty (low vs. high) as within-subject factor. To explore whether *DTE* results were influenced by reachable workspace area, we repeated the analysis of *DTE* using linear mixed models with workspace area as a covariate, with group as between subject factor and with task (cognitive vs. motor) and motor-task difficulty as within-subject factors. We also explored whether *DTE* results were influenced by single-task performance, which is in the denominator of Equation 1. We therefore repeated the analysis of *DTE* using a linear mixed model with single-task performance as a covariate, with group as between subject factor and with task and motor-task difficulty as within-subject factors.

Significance was set at $P < .05$. Significant interaction effects were analyzed using simple effects analyses, which yielded the effect of one independent variable at individual levels of the other independent variable (Field, 2009).

Results

Reachable workspace area as covariate

Results of the ANCOVA on single-task P_M (Table B.1) showed that reachable workspace area contributed significantly to the model, both for PD/control and stroke/control. The current analyses revealed significant main effects of group that were complemented by significant interactions between group and motor-task difficulty. Similar to the findings presented in the main paper, single-task P_M was lower in each of the patient groups compared to controls, for both levels of motor-task difficulty. For all three groups P_M was lower for the high-difficulty compared to the low-difficulty level of the motor task (all $P < .001$). This difficulty effect was more pronounced for controls than for the patient groups.

Results of the linear mixed model on *DTE* (Table B.1) showed that reachable workspace area contributed significantly to the stroke/control model. Effects on interpretation of the *DTE* results were minimal. Similar to the findings presented in the main paper, the current analyses showed that PD patients experienced more interference (i.e., more negative values of *DTE*) than controls, while there was no difference in *DTE* between stroke patients and controls. In both analyses, there were no significant interactions between group and task or motor-task difficulty. Follow-up analyses on the interaction between task and motor-task difficulty yielded largely similar results for analyses based on PD/control and stroke/control. In specific, interference on the cognitive task markedly increased when obstacles were introduced (i.e., more negative DTE_C; PD/control: $P < .001$; stroke/control: $P < .001$) while interference on the motor task tended to decrease (i.e., less negative DTE_M; PD/control: $P =$ .004; stroke/control: $P = .15$). Hence, the high-difficulty motor task was prioritized over the cognitive task (i.e., DTE_C more negative than DTE_M; PD/control: $P < .001$; stroke/control: $P <$

.001), whereas the cognitive task was prioritized over the low-difficulty motor task (i.e., DTE_C less negative than DTE_M; PD/control: P = .02; stroke/control: P = .04).

Table B.1. Reachable workspace area as covariate

		PD versus controls			Stroke versus controls		
**Outcome**	**Effect**	Test statistic		P	Test statistic		P
P_M	RWA	$F_{1,102}$ =	5.47	.02	$F_{1,98}$ =	65.92	<.001
	G	$F_{1,102}$ =	36.92	<.001	$F_{1,98}$ =	10.05	.002
	D	–	–		–	–	
	G × D	$F_{1,102}$ =	10.08	.002	$F_{1,98}$ =	7.50	.007
DTE	RWA	$F_{1,102}$ =	0.17	.68	$F_{1,98}$ =	6.78	.01
	G	$F_{1,102}$ =	14.26	<.001	–	–	
	T	$F_{1,309}$ =	15.29	<.001	$F_{1,297}$ =	5.44	.02
	D	$F_{1,309}$ =	9.56	.002	$F_{1,297}$ =	9.91	.002
	T × D	$F_{1,309}$ =	52.46	<.001	$F_{1,297}$ =	27.19	<.001

Analysis based on n = 56 controls vs. n = 54 PD patients, and on n = 56 controls vs. n = 45 stroke patients. Linear Mixed Model with reachable workspace area (RWA) as covariate; G = group, as indicated; D = motor-task difficulty (low vs. high); T = task (cognitive vs. motor, for *DTE* only).

Single-task performance as covariate

Results of the linear mixed model on *DTE* (Table B.2) showed that single-task performance contributed significantly to model, both for PD/control and stroke/control. Similar to the findings presented in the main paper, the current analysis showed that PD patients experienced more interference (i.e., more negative values of *DTE*) than controls (P < .001), while there was no difference in *DTE* between stroke patients and controls. In both analyses, there were no significant interactions between group and task or motor-task difficulty. Although the main effect of task was no longer significant in the PD/control analysis, the main effect of motor-task difficulty was still complemented by an interaction between task

and motor-task difficulty in both analyses. Follow-up analyses on the interaction between task and motor-task difficulty yielded largely similar results for analyses based on PD/controls and stroke/controls. In specific, interference on the cognitive task markedly increased when obstacles were introduced (i.e., more negative DTE_C; PD/control: P < .001; stroke/control: P < .001) while interference on the motor task tended to decrease (i.e., less negative DTE_M; PD/control: P = .02; stroke/control: P = .62). The high-difficulty motor task tended to be prioritized over the cognitive task (i.e., DTE_C more negative than DTE_M; PD/control: P = .003; stroke/control: P = .60), whereas the cognitive task was prioritized over the low-difficulty motor task (i.e., DTE_C less negative than DTE_M; PD/control: P = .002; stroke/control: P < .001). Findings for PD/control were similar to those presented in the main manuscript. Also findings for stroke/control were largely similar to those presented in the main manuscript, except that higher motor-task difficulty appeared not associated with prioritization of the motor task or, related to this, decreased interference on the motor task.

Table B.2. Single-task performance as covariate

		**PD versus controls**			**Stroke versus controls**		
**Outcome**	**Effect**	Test statistic		P	Test statistic		P
DTE	STP	$F_{1,404}$ =	3.96	.047	$F_{1,371}$ =	18.96	<.001
	G	$F_{1,103}$ =	17.00	<.001	–	–	
	T	–	–		$F_{1,394}$ =	4.43	.04
	D	$F_{1,312}$ =	12.02	<.001	$F_{1,300}$ =	15.29	<.001
	T × D	$F_{1,312}$ =	46.04	<.001	$F_{1,300}$ =	21.28	<.001

Analysis based on n = 56 controls vs. n = 54 PD patients, and on n = 56 controls vs. n = 45 stroke patients. Linear Mixed Model with single task performance as covariate (single-task P_M for DTE_M and single-task P_C for DTE_C); STP = single-task performance; G = group, as indicated; D = motor-task difficulty (low vs. high); T = task (cognitive vs. motor).

Discussion

Although reachable workspace area contributed to the ANCOVA on single-task P_M, it had negligible effect on interpretation of the effects of interest (i.e., group and motor-task difficulty, and their interactions). Similarly, reachable workspace area and single-task performance contributed to the respective linear mixed models on *DTE* but had no significant effect on interpretation of the effects of interest (i.e., group, task, motor-task difficulty and their interactions), except that prioritization of the high-difficulty motor task over the cognitive task was no longer evident in the stroke/control analysis with single-task performance as covariate. Together, these results indicated that most findings as presented in the main paper are not attributable to or distorted by differences in reachable workspace area (and the associated differences in movement distance between the targets) or differences in single-task performance. However, as mentioned in the main manuscript, one should be aware of potential effects of very low single-task performance, which can lead to disproportionally large DTE values with only a small deterioration or improvement of performance under dual-task conditions.

Supporting Information C ─ Correlation with 'fun' and 'difficulty' ratings.

As described in the main paper, we aimed to further our understanding into the factors contributing to cognitive-motor interference. We therefore explored whether attention allocation was related to perceived 'fun' and 'difficulty' of the tasks.

Methods

After completing all conditions, participants rated the perceived 'fun' and 'difficulty' of the cognitive and motor task on 11-point numeric rating scales (0: none, 10: maximum possible). Within each group of participants, Spearman's correlation coefficient was used to explore whether the perceived 'fun' or 'difficulty' of the motor and cognitive task were associated dual-task effects (DTE_{total}, DTE_C and DTE_M) and *Priority*.

Results

All correlation coefficients are presented in Table C.1. In the two patient groups, task prioritization (*Priority*) was not related to difficulty ratings or fun ratings on either the motor task or the cognitive task. Only small but significant correlations were observed between difficulty ratings on the cognitive task and DTEs. In PD patients, higher difficulty ratings were associated with more interference (more negative DTE_{total}) in the high-difficulty dual-task condition, whereas in stroke patients higher difficulty ratings were associated with less interference (DTE_{total} and DTE_M) in the low-difficulty dual-task condition.

In controls, prioritization of the motor task (i.e., negative values of *Priority*) was associated with higher difficulty ratings on the motor task. Notably, only within the control group significant correlations with fun ratings were observed. In particular, higher fun ratings on the cognitive task were associated with higher values of DTE_{total} and DTE_C, indicating less

interference in especially the cognitive domain (regardless of difficulty level of the motor task), and with higher values of *Priority*, indicating cognitive priority. Moreover, higher fun ratings on the motor task were associated with higher values of DTE_{total} and DTE_C, indicating less interference in especially the cognitive domain, but only so for the low-difficulty level of the motor task (i.e., without obstacles).

Table C.1 Correlation with 'fun' and 'difficulty' ratings.

	Cognitive task				Motor task			
	Fun rating		Difficulty rating		Fun rating		Difficulty rating	
Difficulty level	Low	High	Low	High	Low	High	Low	High
PD patients								
DTE_{total}	.04	.24	-.18	-.34*	-.17	-.12	-.15	-.22
DTE_C	.11	.16	-.15	-.23	-.06	-.03	-.09	-.14
DTE_M	-.08	.20	-.14	-.27	-.26	-.19	-.15	-.17
Priority	.18	-.03	.01	.04	.16	.17	.17	-.02
Stroke patients								
DTE_{total}	-.03	.18	.31*	.13	-.07	.11	-.13	-.16
DTE_C	.12	.18	.14	.05	-.01	.09	-.14	-.05
DTE_M	-.18	.11	.36*	.17	-.22	.04	.06	-.04
Priority	.17	.09	-.20	-.03	.04	.01	-.06	.01
Controls								
DTE_{total}	.42**	.30*	-.15	-.24	.32*	.13	-.21	-.11
DTE_C	.43**	.31*	-.15	-.22	.31*	.17	-.19	-.19
DTE_M	.17	.02	-.16	-.10	.12	-.13	-.15	.22
Priority	.34*	.27*	-.11	-.19	.20	.22	-.15	-.28*

* $P < .05$, ** $P < .01$. Note: Fun and difficulty were rated on a scale from 0 (none) to 10 (maximum possible). Negative values of DTE_{total}, DTE_C and DTE_M indicate cognitive-motor interference. Negative values of *Priority* indicate prioritization of the motor task over the cognitive task.

Discussion

With this exploratory analysis we aimed to further our understanding into the factors contributing to cognitive-motor interference. Results showed that in both patient groups fun ratings and difficulty ratings of the two tasks were not consistently related to attention allocation and task prioritization. Only in the control group attention allocation and task prioritization appeared associated with the perceived ‘fun’ and ‘difficulty’ of the tasks. In particular, higher fun ratings on the cognitive task were associated with more prioritization of the cognitive task. Higher difficulty ratings on the motor task were associated with more prioritization of the motor task, but only for the high-difficulty dual-task condition (i.e., with obstacles).

Based on these findings in combination with the findings presented in Table 3 of the main manuscript, it may tentatively be suggested that healthy individuals were more flexible than patients in their attention allocation and task prioritization. It has been suggested that the self-selected strategy of task prioritization is determined by factors that minimize danger and maximize pleasure (Williams, 2006). In line with this, healthy individuals tended to prioritize the more ‘fun’ task when task complexity allowed (i.e., in the low-difficulty condition), whereas in more challenging conditions (i.e., with obstacles) the more difficult motor task was prioritized, perhaps to preserve at least a ‘minimally acceptable level of performance’ or in an attempt to minimize danger. Although decreased performance on the upper-limb motor task would not pose a threat to physical safety, as would have been the case for walking or maintaining balance, it is conceivable that participants considered the suddenly appearing obstacle (i.e., a virtual cat) as threatening to the virtual mouse of which they controlled the vertical and horizontal movements.

Patients with neurological deficits, however, seemed less flexible in their strategy. Performance in dual-task conditions appeared more related to their cognitive and/or motor

abilities than to fun ratings for the respective tasks. For PD patients, reduced cognitive function (i.e., lower scores on the SCOPA-COG) was associated with more interference on the cognitive task (i.e., more negative DTE_C; main manuscript). For stroke patients, impaired motor function (i.e., lower score on the FM-UE) was associated with more dual-task interference (i.e., more negative values of DTE_{total}). These findings underscore that the mediators of dual-task interference appear more complex than “a core motivation to minimize danger and maximize pleasure” (Williams, 2006). In addition to cognitive and motor function, also the cognitive reserve and compensatory abilities, personality, affect and expertise may play a role (Yogev-Seligmann *et al.*, 2012). The self-selected strategy for task prioritization may thus differ between individuals, between different combinations of dual-tasks (e.g., when difficulty of the motor task is increased) and even between measurement sessions (which may have contributed to the low test-retest reliability presented in Supporting Information A). Our findings thus corroborate the recommendation of Plummer and Eskes (2015) to assess performance in both the cognitive and motor domain under single- and dual-task conditions, evaluate DTEs in both domains and determine the pattern of CMI (see Figs. 2 and 4 of the main manuscript) for each individual patient to gain insight into attention allocation as well as overall dual-task capacity, which appears critical for accurate interpretation of CMI and its responses to treatment.

References

Bland, J.M. & Altman, D.G. (1999) Measuring agreement in method comparison studies. *Stat Methods Med Res*, **8**, 135-160.

Cicchetti, D.V. (1994) Guidelines, criteria, and rules of thumb for evaluating normed and standardized assessment instruments in psychology. *Psychol Assess*, **6**, 284.

de Vet, H.C., Terwee, C.B., Knol, D.L. & Bouter, L.M. (2006) When to use agreement versus reliability measures. *J Clin Epidemiol*, **59**, 1033-1039.

Field, A. (2009) *Discovering statistics using SPSS*. Sage publications.

Plummer, P. & Eskes, G. (2015) Measuring treatment effects on dual-task performance: a framework for research and clinical practice. *Front Hum Neurosci*, **9**, 225.

Shoukri, M., Asyali, M. & Donner, A. (2004) Sample size requirements for the design of reliability study: review and new results. *Stat Methods Med Res*, **13**, 251-271.

Williams, L.M. (2006) An integrative neuroscience model of "significance" processing. *J Integr Neurosci*, **05**, 1-47.

Yogev-Seligmann, G., Rotem-Galili, Y., Dickstein, R., Giladi, N. & Hausdorff, J.M. (2012) Effects of explicit prioritization on dual task walking in patients with Parkinson's disease. *Gait Posture*, **35**, 641-646.
