# Supplementary material for: Cognitive‐motor interference during goal‐directed upper‐limb movements
Source: Eur J Neurosci. 2018 Oct 20;48(10):3146–58. doi: 10.1111/ejn.14168 (PMC6282826; doi:10.1111/ejn.14168)
Supplement: Supplementary file 1 [file EJN-48-3146-s002.docx]

Cognitive-motor interference during goal-directed upper-limb movements

|  | Bank, Paulina ; Marinus, Johan; van Tol, Rosanne; Groeneveld, Iris; Goossens, Paula; de Groot, Jurriaan; van Hilten, Jacobus; Meskers, Carel |
| --- | --- |

_____________________________________________________________________________________

| Review timeline: | Submission date: 27/03/18  Editorial Decision: Major Revision  Revision Received: 04/07/18  Editorial Decision: Minor Revision  Revision Received: 03/09/18  Accepted: 11/09/18 |  |
| --- | --- | --- |
|  |  |  |

Editor: Ali Mazaheri

Reviewer 1: Rick Helmrich

_____________________________________________________________________________________

1st Editorial Decision	18/06/18

_____________________________________________________________________________________

Cognitive-motor interference during goal-directed upper-limb movements

Dear Dr. Bank,

First of all, apologies for the time it has taken to deal with your manuscript.  this was due to difficulty in engaging reviewers.  In view of this we have decide to go ahead with a single reviewer as well as comments from the Section Editor,  Dr. Ali Mazaheri.

The review, and our thoughts, collectively indicate that your experiments generated new and important information.  However, there are several issues that need to be clarified/resolved before we can consider your manuscript further for publication in EJN.

As you can see, the reviewer raises one substantial criticism which pertains to why you would group the stroke patients with the controls, to compare to the PD patients.  
The suggestion of the reviewer was to either compare PD and controls separate from stroke, or come up with a rational for why you are comparing these two very distinct disorders and the heterogeneity of the stroke group.

We also noted the following points that need to be addressed in the revised version.
- The abstract doesn't motivate the study, please state why the study was performed.
- The English needs some improvement, please carefully proof-read.
- Reference list: Geijtenbeek et al entry is missing the publication year
- Higher resolution figures will be needed for publication.
- In accordance with EJN guidelines, please replace the bar charts with more informative scatter plots, hybrid plots or similar.  
- Appendices should be separated from the main text file and moved to supplementary material  

When revising the manuscript, please embolden or underline major changes to the text so they are easily identifiable and DO NOT leave 'track change' formatting marks in your paper.  Please ensure that you provide a text and a figure file for the Graphical Abstract (as detailed in the instructions below).  When carrying out your revisions please refer to the checklist below and visit the EJN author guidelines at www.ejneuroscience.org

When finalized, please upload your complete revised manuscript onto the website, as a Word file (.doc, or .docx).  Please also ensure that a complete set of tables and figures is included as separate files, even if these have not changed from the originals.  At this stage it is necessary to provide high resolution figures.  Please see important instructions below.

Please go into https://mc.manuscriptcentral.com/ejn - Author Centre - manuscripts with decisions where you will find a 'create a revision' link under 'actions'. We ask that you please indicate the way in which you have responded to the points raised by the Editors and Reviewers in a letter. Please upload this response letter as a separate Word (.doc or PDF) file using the file designation "Authors' Response to Reviewers" when uploading your manuscript files.  Please DO NOT submit your revised manuscript as a new one.  Also, please note that only the  Author who submitted the original version of the manuscript should submit a revised version.

If you are able to respond fully to the points raised, we would be pleased to receive a revision of your paper within 12 weeks.

Thank you for submitting your work to EJN.

Best wishes,

Paul Bolam & John Foxe
co-Editors in Chief, EJN


Reviews:

Reviewer: 1

Comments to the Author
In this behavioral study, the authors investigated cognition-motor interference (CMI) in two neurological diseases: Parkinson's disease and stroke. They confirmed previous reports that Parkinson patients had abnormal CMI compared to controls. Stroke patients were not different from controls.

This study has large samples (n=57 in each patient group). On the other hand, the question addressed is not very novel (many previous studies have reported dual task interference in PD with slightly different paradigms).

My main critique is that it is not clear why the authors combined PD and stroke patients into a single study, even within a single statistical analysis. It appears to me they are comparing apples and oranges, and there is no clear rationale for this comparison in the introduction. Furthermore, stroke have very different clinical phenotypes, depending on the size and the location of the stroke. The authors included patients with different types of stroke (left/right, ischemic/hemorrhage, different locations). I wonder what this heterogeneous sample tells us about stroke, or about the functioning of the healthy brain. Maybe this heterogeneity explains why the authors did not find clear effects in the stroke group. My advise would be to either separate the analyses comparing PD versus controls from those comparing stroke versus controls, or to better present the rationale for comparing the two disorders.

____________________________________________________________________________________

Authors’ Response	04/07/18

_____________________________________________________________________________________

Please find enclosed the revised version of our manuscript entitled **“Cognitive-motor interference during goal-directed upper-limb movements”**. We have addressed the comments of the reviewer and the section editor (see our point-by-point reply on the following pages) and revised the manuscript accordingly. We would like to thank the reviewer and the section editor for the constructive comments, which helped us to further improve our manuscript.

**Reviewer #1:**

In this behavioral study, the authors investigated cognition-motor interference (CMI) in two neurological diseases: Parkinson's disease and stroke. They confirmed previous reports that Parkinson patients had abnormal CMI compared to controls. Stroke patients were not different from controls.

This study has large samples (n=57 in each patient group). On the other hand, the question addressed is not very novel (many previous studies have reported dual task interference in PD with slightly different paradigms).

My main critique is that it is not clear why the authors combined PD and stroke patients into a single study, even within a single statistical analysis. It appears to me they are comparing apples and oranges, and there is no clear rationale for this comparison in the introduction. Furthermore, stroke have very different clinical phenotypes, depending on the size and the location of the stroke. The authors included patients with different types of stroke (left/right, ischemic/hemorrhage, different locations). I wonder what this heterogeneous sample tells us about stroke, or about the functioning of the healthy brain. Maybe this heterogeneity explains why the authors did not find clear effects in the stroke group. My advise would be to either separate the analyses comparing PD versus controls from those comparing stroke versus controls, or to better present the rationale for comparing the two disorders.

*In our study, we developed a protocol for evaluating CMI during upper-limb motor control. We used it to evaluate DTEs in both the motor and cognitive domain in healthy individuals and in two highly prevalent neurological disorders that are associated with (varying degrees of) deficits of cognitive and/or motor processing (PD and stroke). These patient groups were chosen as ‘proof of concept’ since they were expected to show increased levels of CMI that may depend on the severity of cognitive and/or motor symptoms. We originally combined these groups into a single statistical analysis with planned comparisons (PD vs. control and stroke vs. control) to evaluate significant group effects. We deliberately refrained from formulating a priori hypotheses on differences between PD and stroke on CMI and hence, we agree with the reviewer that it is more appropriate to conduct two separate analyses: one for comparing PD patients versus controls and one for comparing stroke patients versus controls. We have re-run the statistical analysis and have revised the manuscript accordingly (main manuscript and Supporting Information B). In view of readability, we have presented the statistical test results of significant main and interaction effects in a table (Table 2, separate columns for PD patients vs. controls and for stroke patients vs. controls). Main findings and results of associated post-hoc analyses are described in the text. In order to avoid repetition and to preserve readability, we do not provide strictly separated descriptions of results for PD/control and stroke/control (e.g. in separate sections) but combined where possible. For example on p. 15, post-hoc results for the task and motor-task difficulty on DTE are described simultaneously for both the PD/control and stroke/control analysis because no significant interaction effects with group were observed and post-hoc analyses of the interaction between task and motor-task difficulty on DTE yielded similar results for both analyses.*

*With respect to the reviewer’s comment about heterogeneity, we would like to point out that we included patients with varying degree of motor and cognitive impairments on purpose, and that the implications of this heterogeneity are mentioned in our manuscript (discussion, p. 17-18 and 20, see below). Importantly, the variation in severity of cognitive and motor impairments within each patient group, as well as variation in the outcome parameter, allowed us to evaluate the association between the severity of cognitive and/or motor impairments and CMI. We agree with the reviewer that heterogeneity of especially the stroke population may have interfered with the identification of differences in DTE patterns and we acknowledged this issue in the manuscript. However we do feel that we can elaborate on differences between PD and stroke versus controls and even between PD and stroke as we do in the discussion section. Note that the heterogeneity is more outspoken for the motor impairments and with regard to DTE_M_ (i.e., larger dispersion along the y-axis of Fig. 4). Together with the finding that the group of stroke patients did experience problems with the cognitive and motor task under single-task conditions (Figure 3) we note that at least for the less affected stroke patients CMI may play a less dominant role than for PD patients.*

*Furthermore, as stated on p.20, our study was not intended to find the specific brain areas involved in cognitive-motor interference: because of the heterogeneity in terms of stroke type and location, our stroke patients would not be the appropriate subjects to study the involvement of a specific brain area. For the current study, however, it is of particular importance that all stroke patients were tested >8 weeks after stroke and had residual motor impairments (to a varying degree) whether or not accompanied by cognitive impairments (to a varying degree).*

*The implications of the heterogeneity within our patient groups are addressed in the discussion as follows:*

*p. 17-18	“In contrast to our expectations, stroke patients in general did not experience greater CMI than controls. Substantial heterogeneity within this patient group (in terms of lesion location and severity of cognitive and motor impairments) may have played a role in this regard”.*

*p. 20	“Compared to previous works, our study has some important advantages. Firstly, our study includes a large number of participants with a varying degree of cognitive and motor impairments, which allowed us to not only compare CMI between patients and controls, but also to explore the associations between DTEs and clinical tests of cognitive and motor function.”*

*p. 20	“Firstly, our study was not intended to find the specific brain areas involved in CMI. This would require a more homogenous stroke population in terms of location of the lesion.”*

**Section editor:**

As you can see, the reviewer raises one substantial criticism which pertains to why you would group the stroke patients with the controls, to compare to the PD patients. The suggestion of the reviewer was to either compare PD and controls separate from stroke, or come up with a rational for why you are comparing these two very distinct disorders and the heterogeneity of the stroke group.

*In our study we developed a protocol to evaluate CMI in healthy individuals and in two highly prevalent neurological disorders that are associated with (varying degrees of) deficits of cognitive and/or motor processing (PD and stroke). We chose these distinct patient groups as ‘proof of concept’ since they were expected to show increased levels of CMI that may depend on the severity of cognitive and/or motor symptoms. We originally combined these groups into a single statistical analysis with planned comparisons (PD vs. control and stroke vs. control) to follow up on significant effects of group. However, we agree with the reviewer that it is more appropriate to conduct separate analyses: one for comparing PD patients versus controls and one for comparing stroke patients versus controls. In response to the reviewer’s comment we have therefore re-run the statistical analyses and have revised the manuscript accordingly (main manuscript and Supporting Information B). Effects on interpretation of our results were negligible.*

*With respect to the heterogeneity of the stroke group, it should be noted that our study was not intended to find the specific brain areas involved in cognitive-motor interference (p. 20). Indeed, because of the heterogeneity in terms of stroke type and location, our stroke patients would not be the appropriate subjects to study the involvement of a specific brain area. For the current study, it is of particular importance that all stroke patients had residual motor impairments (to a varying degree) whether or not accompanied by cognitive impairments (to a varying degree). We included patients with varying degree of motor and cognitive impairments on purpose: the variation in severity of cognitive and motor impairments within each patient group, as well as variation in the outcome parameter, allowed us to evaluate the association between the severity of cognitive and/or motor impairments and CMI. At the same time, we acknowledge the fact that the heterogeneity of the patient population may have interfered with the establishment of differences in DTE (as outlined in the discussion section on pp. 17-18 and 20). Nevertheless, we do feel that this issue does not refrain us from elaborations on differences in CMI between groups.*

We also noted the following points that need to be addressed in the revised version.

- The abstract doesn't motivate the study, please state why the study was performed.

*We have added a brief motivation to the abstract. The text in the abstract now reads:*

*“Research and clinical practice have focused on effects of a cognitive dual-task on highly automated motor tasks such as walking or maintaining balance. Despite potential importance for daily life performance, there are only a few small studies on dual-task effects on upper-limb motor control. We therefore developed a protocol for assessing cognitive-motor interference (CMI) during upper-limb motor control and used it to evaluate dual-task effects in 57 healthy individuals and two highly prevalent neurological disorders associated with deficits of cognitive and motor processing (57 patients with Parkinson’s disease [PD], 57 stroke patients).”*

- The English needs some improvement, please carefully proof-read.

*We have carefully proof-read the manuscript and revised several sentences. We are now confident that the English is of sufficient quality.*

- Reference list: Geijtenbeek et al entry is missing the publication year.

*Thank you for pointing this out. We have added the missing information.*

- Higher resolution figures will be needed for publication.

*We have provided higher-resolution figures.*

- In accordance with EJN guidelines, please replace the bar charts with more informative scatter plots, hybrid plots or similar.

*We have added the individual data points to Figure 3. In view of the complexity of the figure (especially Figure 3C), we decided to keep the bars and error bars (representing mean values and SE) next to the individual data points. This contributes greatly to the clarity of the figure and helps the readers better understand (changes in) the relationship between DTE_C_ and DTE_M_.*

- Appendices should be separated from the main text file and moved to supplementary material

*Done*

2^nd^ Editorial Decision	28/08/18

_____________________________________________________________________________________

Cognitive-motor interference during goal-directed upper-limb movements

Dear Dr. Bank,

Your revised manuscript was re-evaluated by external reviewers as well as by the Section Editor, Dr. Ali Mazaheri and ourselves.  We are pleased to inform you that we expect that it will be acceptable for publication in EJN following further minor revision.   The revised manuscript will be dealt with by the editors.

We commend the efforts you have made revising the manuscript and believe that it represents an important contribution to the field.   The reviewer still holds on to one small issue, which we would like you to attempt address. Could you put in the introduction a  small paragraph outlining your motivation and rational for combining both patient groups?

In addition:
- Please remove author qualifications form the author list.
- Could you make the 'Competing interests' statement into a sentence. 'The authors have no competing interests'?
- We will need higher resolution figures for publication (600 x 600 dpi).

When revising the manuscript, please embolden or underline major changes to the text so they are easily identifiable and please don't leave 'track change' formatting marks in your paper.  At this stage, please provide text and a figure file for the Graphical Abstract.  When carrying out your revisions please refer to the checklist below and visit the EJN author guidelines at www.ejneuroscience.org

When finalised, please upload your complete revised manuscript onto the website as a Word (.doc or .docx), or .rtf file.  Please also ensure that a complete set of tables and figures is included as separate files, even if these have not changed from the originals.  At this stage it is necessary to provide high resolution figures.  Please see important instructions below.

Please go into https://mc.manuscriptcentral.com/ejn - Author Centre - manuscripts with decisions where you will find a 'create a revision' link under 'actions'. We ask that you please indicate the way in which you have responded to the points raised by the Editors and Reviewers in a letter. Please upload this response letter as a separate Word (.doc) file using the file designation "Authors' Response to Reviewers" when uploading your manuscript files. Please DO NOT submit your revised manuscript as a new one.  Also, please note that only the  Author who submitted the original version of the manuscript should submit a revised version.

If you are able to respond fully to the points raised, we shall be pleased to receive a revision of your paper within 30 days.

Thank you for submitting your work to EJN.

Best wishes,

Paul Bolam & John Foxe
co-Editors in Chief, EJN

Reviews:

Reviewer: 1

Comments to the Author
The authors have addressed my main comment, and have now separated the statistical analyses for the Parkinson patients (versus controls) and the stroke patients (versus controls). I still question the usefulness of combining both patient groups in this paper; the rationale is still not clear to me. Also the novelty is modest. Nevertheless, the methods appear sound, and as such I do not see a reason for rejection.

_____________________________________________________________________________________

Authors’ Response	03/09/18

_____________________________________________________________________________________

Please find enclosed the revised version of our manuscript entitled **“Cognitive-motor interference during goal-directed upper-limb movements”**. We have addressed all remaining comments (see our point-by-point reply on the following pages) and revised the manuscript accordingly.

**Reviewer 1:**

The authors have addressed my main comment, and have now separated the statistical analyses for the Parkinson patients (versus controls) and the stroke patients (versus controls). I still question the usefulness of combining both patient groups in this paper; the rationale is still not clear to me. Also the novelty is modest. Nevertheless, the methods appear sound, and as such I do not see a reason for rejection.

*We are pleased that the reviewer is satisfied with our responses and the revisions to our manuscript. As requested by the editor (see below), we have added a short paragraph to the introduction, outlining our motivation for including the two patient groups.*

**Editor:**

We commend the efforts you have made revising the manuscript and believe that it represents an important contribution to the field. The reviewer still holds on to one small issue, which we would like you to attempt address. Could you put in the introduction a small paragraph outlining your motivation and rational for combining both patient groups?

*Thank you for your positive response to our manuscript. As extensively motivated in our previous response to the reviewer’s comment, we chose the two patient groups in our study as ‘proof of concept’ because 1) they are highly prevalent neurological conditions, 2) they are associated with deficits of cognitive and motor processing, 3) they are expected to show increased levels of CMI, and 4) because the considerable variation in severity of cognitive and motor impairments within these patient groups would allow evaluation of the association between the severity of cognitive and/or motor impairments and (patterns of) CMI. As requested, we have added a small paragraph to the introduction, outlining our motivation for including the two patient groups.*

*The revised paragraph in the introduction (p. 5) now reads:*

*“ In this study, we therefore developed a protocol for evaluating patterns of CMI during simultaneous performance of a cognitive task and an upper-limb motor task. We used it to evaluate DTEs in both the motor and cognitive domain in healthy individuals and in two highly prevalent neurological conditions associated with deficits of cognitive and motor processing (PD and stroke). These distinct patient groups were chosen as a generalized ‘proof of concept’ because they were expected to show increased levels of CMI and because the considerable variation in severity of cognitive and motor impairments within these patient groups would allow evaluation of the association between the severity of cognitive and/or motor impairments and (patterns of) CMI.”*

In addition:

- Please remove author qualifications form the author list.

*Done.*

- Could you make the 'Competing interests' statement into a sentence. 'The authors have no competing interests'?

*Done.*

- We will need higher resolution figures for publication (600 x 600 dpi).

*We have provided high-resolution figures (all figures have a resolution of 600 x 600 dpi).*
